# Effects of Zerovalent Iron Nanoparticles on Photosynthesis and Biochemical Adaptation of Soil-Grown *Arabidopsis thaliana*

**DOI:** 10.3390/nano9111543

**Published:** 2019-10-30

**Authors:** Hakwon Yoon, Yu-Gyeong Kang, Yoon-Seok Chang, Jae-Hwan Kim

**Affiliations:** 1 Division of Environmental Science and Engineering, Pohang University of Science and Technology (POSTECH), Pohang 37673, Korea; hwyoon@postech.ac.kr (H.Y.);; 2 Advanced Geo-materials R&D Department, Pohang Branch, Korea Institute of Geoscience and Mineral Resources (KIGAM), Pohang 37559, Korea

**Keywords:** nanoscale zerovalent iron (nZVI), photosynthesis, plant, biochemical response, nutrient

## Abstract

Nanoscale zerovalent iron (nZVI) is the most widely used nanomaterial for environmental remediation. The impacts of nZVI on terrestrial organisms have been recently reported, and in particular, plant growth was promoted by nZVI treatment in various concentrations. Therefore, it is necessary to investigate the detailed physiological and biochemical responses of plants toward nZVI treatment for agricultural application. Here, the effects of nZVI on photosynthesis and related biochemical adaptation of soil-grown *Arabidopsis thaliana* were examined. After treatment with 500 mg nZVI/kg soil, the plant biomass increased by 38% through enhanced photosynthesis, which was confirmed by the gas-exchange system, carbon isotope ratio and chlorophyll content analysis. Besides, the iron uptake of the plant increased in roots and leaves. The magnetic property measurements and transmission electron microscopy showed that the transformed particles were accumulated in parts of the plant tissues. The accumulation of carbohydrates such as glucose, sucrose and starch increased by the enhanced photosynthesis, and photosynthetic-related inorganic nutrients such as phosphorus, manganese and zinc maintained homeostasis, according to the increased iron uptake. These findings suggest that nZVI has additional or alternative benefits as a nano-fertilizer and a promoter of CO_2_ uptake in plants.

## 1. Introduction

Engineered nanomaterials (ENMs) are applied in various fields of industry, such as environmental remediation and agriculture, as well as electronics, catalysts, energy and medical engineering [1,2]. Nanoscale zero-valent iron (nZVI), one of the most dominant ENMs in the environmental industry, was extensively used for its outstanding effectiveness in the remediation of contaminated groundwater [3]. Recently, nZVI was applied for the remediation of soils contaminated with pesticides or heavy metals, thereby improving soil quality [4]. Therefore, the impacts of nZVI on the terrestrial ecosystem should be considered.

Several studies have quantified the effects of nZVI on plants. *Arabidopsis thaliana* exposed to nZVI triggered root elongation, possibly in response to the unique redox properties of nZVI [5]. Low concentrations (≤500 mg/L) of nZVI can increase the biomass of several plant species such as peanut, rice and perennial ryegrass [6,7,8]. Phosphate-sorbed nZVI has also been developed to benefit spinach growth as a phosphate fertilizer and/or iron (Fe) fortifier [9]. On the other hand, high concentrations (>1000 mg/L) of nZVI inhibited the growth of cattail, hybrid poplars and rice [10,11]. As a consequence, an optimal concentration of nZVI might be needed to promote plant growth. However, the effects of nZVI on plants are species-dependent and the reasons that lead to an increase of the biomass or cause toxicity in plants are unclear. Thus, before nZVI can be applied in agriculture, the physiological and biochemical responses of plants to nZVI exposure should be understood. Furthermore, most of the previous studies were conducted in hydroponic systems and not in soil. Instead, a soil system should be used to properly simulate the interaction between plants and nZVI in the ecosystem.

Through photosynthesis, plants play a major role in the terrestrial environment because of their impact on the food supply and climate change. However, there is a forecast that plants on the Earth are inadequate to prevent global warming [12]. Therefore, several studies are being conducted to improve photosynthetic efficiency and crop productivity through genetic modification [13]. In addition to biotechnology, attempts have been made to increase the photosynthetic efficiency through the reactions of “pseudo-transgenic” plants with nanomaterials. Our previous study showed that *Arabidopsis thaliana* exposed to nZVI triggered high plasma membrane (PM) H^+^-ATPase activity and the overexpression of the *AHA2* gene [14]. PM H^+^-ATPase is an essential enzyme for various physiological processes in plants, including nutrient uptake, cell expansion and stomatal control. Therefore, the overexpression of PM H^+^-ATPase promotes stomatal opening, which facilitates CO_2_ uptake, thereby enhancing photosynthesis [15]. Furthermore, PM H^+^-ATPase-related gene (*CsHA1*) in cucumber (*Cucumis sativus*) was overexpressed by nZVI, affecting plant growth and Fe uptake [16]. The exposure to nZVI is assumed to mediate a plant’s activation of the enzyme for stomatal control, but the mechanism of the biochemical response remains to be determined.

The aim of this research is to analyze the effects of nZVI on several aspects of photosynthesis, using soil-grown *Arabidopsis thaliana* as a model species. Even with the increase of studies regarding nZVI effects on plants, there is still much to be done to quantify the extent of the effects of nZVI on plant growth and nutrients and on the photosynthesis involved in their metabolism. The following biomarkers were used to assess the effects of nZVI on photosynthesis: biomass, leaf area, gas-exchange parameters, carbon isotope ratio, chlorophyll, and photosynthesis-related nutrients including mineral elements, sugar, starch and protein. Additionally, the uptake and intracellular distribution of Fe in *Arabidopsis* were observed. Our results indicate that nZVI can promote plant growth by increasing its photosynthesis and nutrient accumulation. These observations raise the possibility of using nZVI as an ecologically benign alternative nano-fertilizer and promoter of CO_2_ uptake.

## 2. Materials and Methods

### 2.1. nZVI Particles

**Figure 1:**
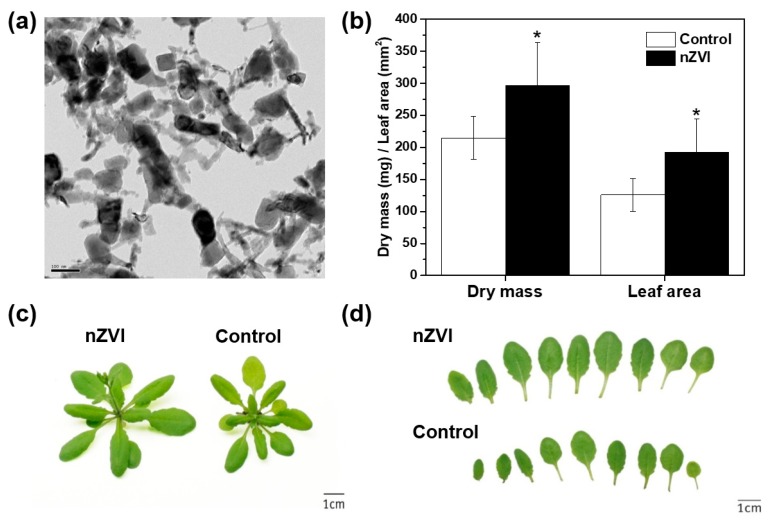
(**a**) TEM image of nanoscale zerovalent iron (nZVI; RNIP-10DS). (**b**) Rosette dry weight and leaf area of 21 d *Arabidopsis* plant observed in two assays (*n* = 12). (**c**,**d**) Phenotype and photo of growth of control and nZVI-exposed *Arabidopsis* shoots at 21 days. The error bar represents standard deviation. The difference was detected by Student’s *t*-test (* *p* < 0.05).

Commercial nZVI (RNIP-10DS, purchased from Toda Kogyo Corp., Tokyo, Japan) was used as representative of nZVI. It has been characterized previously [5,14]. Its Brunauer–Emmett–Teller (BET) surface area, determined by means of a particle size analyzer (UPA-150, Microtrac, Montgomeryville, PA, USA), was 30 ± 2 m^2^·g^−1^, and the mean particle size was 54 ± 1 nm. The weight percent of Fe^0^ in RNIP was 40 ± 3%. The nZVI morphology, characterized by transmission electron microscopy (TEM), is provided in Figure 1a.

### 2.2. Soil Culture and Plant Growth

The methods used for plant growth and nZVI application to the soil were similar to those described previously [5,14,16]. nZVI particles were washed with ethanol, sonicated using a Vibra-Cell sonicator (50 W, frequency 20 KHz, VC50; Sonics and Materials, Inc., Newtown, CT, USA) for 15 min, then rinsed with degassed and deionized (DI) water. This slurry of nZVI was added to 100 g of bed soil (Hungnong Seed Co., Pyeongtaek, Korea). The physicochemical properties of this soil (pH, electrical conductivity (EC), cation exchange capacity (CEC), total organic carbon (TOC) and other nutrient analyses) are described in Table 1. The final concentration of nZVI in the soil was 500 mg·kg^−1^. This dosage was chosen to maximize plant growth by referring to a previous study [14]. The nZVI concentration was also chosen to test the possible effects of a fairly high concentration of nZVI in the soil. *Arabidopsis thaliana* (Colombia ecotype) was cultivated in the plant growth chamber (DS-330DHL, Daewon Sci., Bucheon, Korea) at a controlled temperature (24/22 °C day/night cycle) and 60% relative humidity set to a 16 h photoperiod. The soil moisture was maintained at 60–70%. After 21 days, growth was halted and the weight of the harvested plants was quantified before they flowered. The biomass was measured on the shoots, excluding the roots of plants. Root biomass was not considered in this study because the fine roots of *Arabidopsis* could not be collected completely. Then, the leaves were dried, pressed and photographed against a white background. The leaf area was calculated by image analysis using ImageJ software.

### 2.3. Photosynthetic Capacity Measurement

#### 2.3.1. Gas-Exchange Measurements

The CO_2_ assimilation rate, intracellular CO_2_ concentration, transpiration rate and stomatal conductance were measured using an LI-6400 gas-exchange system (Li-Cor Inc., Lincoln, NE, USA) fitted with a 6400-15 extended-reach 1 cm chamber [15]. The factors related to photosynthesis were kept constant: temperature (22–24 °C), relative humidity (50–60% (Pa/Pa)), pressure (1 atm), light intensity (200 µmol∙m^−2^∙s^−1^), flow rate (500 µmol∙s^−1^) and CO_2_ concentration (400 µL∙L^–1^). All parameters were calculated with software provided by the manufacturer.

#### 2.3.2. Carbon Isotope Ratio Analysis

A carbon isotope analysis was conducted using a stable isotope ratio mass spectrometer (Optima; Micromass Ltd., Wythenshawe, Mancherster, UK) at the Korea National Instrumentation Center for Environmental Management (Seoul, Korea). The dried leaves from 21 d growth rosettes were used for the analysis. The carbon isotope ratio (δ^13^C, ‰) was obtained in δ-notation, where R_sample_, R_standard_, and δ (R_sample_/R_standard_ − 1) are the isotope ratios of the plant sample and the standard (Pee dee belemnite), respectively [15,17].

#### 2.3.3. Chlorophyll Measurement

The plant extracts were prepared by treating leaf tissue with 50 mL of 95% ethanol for 20 min at 80 °C. The amounts of chlorophyll *a* and *b* were calculated using a UV/Vis spectrophotometer at wavelengths of 663 nm and 645 nm, respectively [18].

### 2.4. Determination of Iron and Other Mineral Nutrients

The soil, roots, and shoots were used to determine the total Fe content. The shoots were also sampled to quantify some of the inorganic nutrients essential for photosynthesis (magnesium (Mg), phosphorus (P), zinc (Zn), and manganese (Mn)). The root and shoot tissues were prewashed thoroughly with DI water and calcium chloride (CaCl_2_) solution 3 times. The CaCl_2_ solution minimizes analytical errors through ion exchange of Ca and Fe at the sample’s surface [16,19]. The processed plant and soil samples were dried at 70 °C for 3 d, then weighed. The dried samples were dissolved in 60% HNO_3_ and 30% H_2_O_2_ at 105 °C overnight. After diluting the nitric acid mixture, the elemental contents were measured by inductively coupled plasma optical emission spectrometry (ICP-OES; iCAP6300 DUO, Thermo Scientific, Waltham, MA, USA) [16]. The total mineral nutrient contents were calculated using the USEPA SW-846 method [20]. A superconducting quantum interference device (SQUID) magnetometer (MPMS-5, Quantum Design, San diego, CA, USA) was also used for magnetic analysis to determine the uptake and translocation of nZVI into the plants [16].

### 2.5. Microscopic Observation

The fresh plant samples were washed and incubated in fixation buffer (2% glutaraldehyde and 2% paraformaldehyde in 0.05 M sodium cacodylate, pH 7.2). Post-fixation was performed in 1% osmium tetroxide in sodium cacodylate buffer for 2 h at 4 °C. The samples were stained using 0.5% uranyl acetate, then dehydrated in ethanol and embedded in Spurr’s resin. The samples were sectioned in an ultramicrotome (MT-X, RMC Inc., Tucson, AZ, USA) and restained with uranyl acetate 2% and Reynold’s lead citrate [16,21]. Each section was visualized using field emission high-resolution transmission electron microscopy (JEM-2100F HR-TEM; JEOL Inc., Tokyo, Japan) coupled with energy dispersive spectroscopy (EDS).

### 2.6. Measurement of Soluble Sugar, Starch, and Protein Content

The photosynthesis-related soluble sugar and starch were measured to compare control and nZVI-exposed plants. Soluble sugars were extracted from the frozen powder of the plants. Further, 75% (v/v) ethanol was added to the powder, which was then incubated in a sonicator for 1 h. The supernatant was filtered through a 0.2 μm membrane filter, then loaded for high-performance liquid chromatography (HPLC) (Dionex Ultimate 3000, Sunnyvale, CA, USA) with a Shodex RI-101 detector [22]. The HPLC conditions were as follows: a Sugar-Pak (Waters, Milford, MA, USA) 6.5 mm × 300 mm column was used, the mobile phase was distilled water, the flow rate was 0.5 mL min^−1^, the temperature was 70 °C and the injection volume was 10 μL. The sucrose and glucose standards were injected as references. The total starch was determined using a starch assay kit (SA-20; Sigma-Aldrich, St. Louis, MO, USA) [23]. The protein content was quantified as in Zhao et al. [24]. Specifically, 1 g of frozen shoot powder was extracted with 2 mL of QB buffer (adding 5% (v/v) of 2 M KPO_4_ (pH 7.8), 0.2% (v/v) of 0.5 M EDTA, 1% (v/v) of Triton X-100, 12.5% (v/v) ml of 80% glycerol and 81.1% (v/v) of water). Immediately before analysis, 100 µL of 1.0 M of dithiothreitol was added. The extracted solutions were centrifuged for 20 min at 16,000× *g* at 4 °C. The supernatants were used for BCA protein assay.

## 3. Results and Discussion

### 3.1. Effects of nZVI on Plant Biomass

The nZVI treatment clearly affected the phenotype of the *Arabidopsis* shoots (Figure 1b–d). nZVI-exposed plants had 38% higher rosette dry weight and 53% larger leaf area compared to the controls (nontreated wild type). These results indicate that the presence of nZVI in soil benefits shoot growth. As mentioned earlier, several studies reported the stimulation of plant seedling development and growth by nZVI in a hydroponic system. Additionally, the oxidation on nZVI can produce FeO nanoparticles (NPs), such as magnetite (Fe_3_O_4_) and hematite (Fe_2_O_3_), which have nontoxic or positive effects on ryegrass, pumpkin, lettuce and wheat growth [25,26,27]. This benefit may occur because Fe NPs provide bioavailable Fe as a nutrient, or they increase phytohormone content and antioxidant enzyme activity, but the mechanism has not been identified yet [28]. Additionally, the plant response to nZVI may vary depending on the plant species and the physicochemical properties of nZVI, such as aging, size and morphology of NPs [29,30]. To best of the authors’ knowledge, this is the first observation of the stimulation of plant growth by nZVI treatment in the soil system.

### 3.2. Impact of nZVI on Photosynthetic Activity

The dynamic of gas exchange was monitored at 21 d in soil-grown plants amended with nZVI. The gas-exchange status of all parameters measured (CO_2_ assimilation rate, intracellular CO_2_ concentration, transpiration rate, and stomatal conductance) was significantly higher for nZVI-exposed plant leaves than control plants (Table 2). This facilitation of gas exchange by stomatal opening is among the most essential processes in plant photosynthesis and transpiration [15]. Especially, increased stomatal conductance has been demonstrated in hydroponics [14], but it should be noted that the same effects were observed in this soil system. However, previous studies have reported several cases in which metal oxide NPs, such as CeO, ZnO, and CuO NPs, reduced gas-exchange dynamics, thus adversely affected plant growth [31], possibly by disrupting the energy transfer or oxidation from the photosystem to the Calvin cycle [32]. To be specific, NPs either boost photosynthesis processes by improving the light harvesting complex in plants or hinder the pathways by blocking the electron transport chain [33]. In addition, the concentration and size of NPs play specific roles in photosynthesis [34]. Therefore, these results indicate that nZVI-mediated stomatal opening in plants contributes to increased photosynthesis. Similar results have been found with transgenic plants, where increasing stomatal conductance also increased photosynthesis and growth [35].

**Figure 2:**
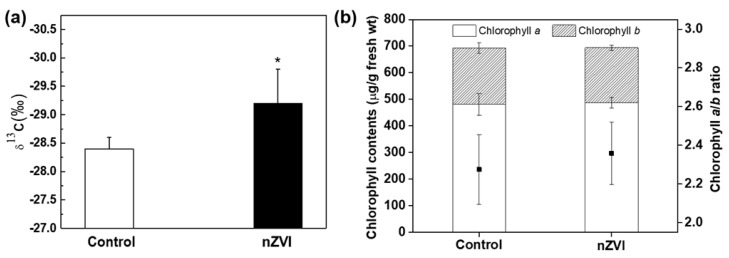
(**a**) Carbon isotope ratio (δ^13^C) and (**b**) chlorophyll content in control and nZVI-exposed *Arabidopsis* shoots at 21 days. Error bar represents standard deviation (*n* = 4). Differences were detected by Student’s *t*-test (* *p* < 0.05).

The carbon isotope ratio (δ^13^C) was measured to evaluate whether the stomatal opening was related to carbon fixation. The nZVI-exposed plants had significantly lower δ^13^C than control plants (Figure 2a). In plants, this number is always negative, which means that ^13^C is less common than ^12^C in the atmosphere [36]. The plants with higher stomatal conductance fix more light ^12^C than heavy ^13^C, therefore low δ^13^C is evidence of high stomatal conductance [37]. Therefore, this result was also indirect evidence that nZVI treatment increases stomatal conductance and thereby increases CO_2_ assimilation in plants from the atmosphere. Transgenic *Arabidopsis* plants, by overexpressing H^+^-ATPase, showed increased photosynthetic activity and plant growth [15]. Although the nZVI-exposed plants had wider stomatal conductance than wild-type plants, they showed identical drought response under conditions of both normal humidity and dehydration [14]. Thus, these results demonstrate that stomatal conductance is an important factor in photosynthesis and is useful to promote plant growth.

Chlorophyll is the major photosynthetic pigment in plants and is sensitive to environmental stress. The chlorophyll content in leaves was measured as another indicator of photosynthetic efficiency. The results of total chlorophyll content indicate no effect of nZVI on photosynthesis by *Arabidopsis* (Figure 2b). In part, the total amounts of chlorophyll *a* and *b* were not significantly different from those of the control group, so the chlorophyll *a*/*b* ratio was also not statistically significant. The Fe deficiency in plants can lead to yellowing of leaves (chlorosis) [38]. Previous studies showed that CeO NPs interfere with the absorption of Fe from the growth medium, resulting in decreased chlorophyll and consequent inhibition of plant growth and photosynthesis [39]. In this study, Fe was sufficiently absorbed into plants (this is discussed in the next section), so the inhibition of chlorophyll synthesis or decreasing photosynthetic efficiency by nZVI was not observed. Even alfalfa grown in nZVI-amended soil contained more chlorophyll than nontreated and Fe-EDTA–treated groups [40]. The magnetized Fe NPs increased chlorophyll content, possibly by influencing both biochemical and enzymatic activity during photosynthesis [41]. Accordingly, nZVI-exposed plants can be proposed as an environmentally benign alternative for reducing atmospheric CO_2_ to mitigate climate change. However, before actual application, the effects of CO_2_ concentration on photosynthesis and the growth of nZVI-exposed plants should be determined.

### 3.3. Effects of nZVI on Nutrient Composition

#### 3.3.1. Organic Nutrients

**Figure 3:**
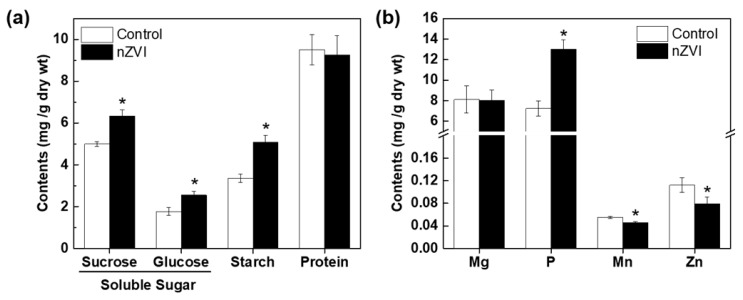
(**a**) Organic nutrients and (**b**) mineral nutrients in control and nZVI-exposed *Arabidopsis* shoot at 21 days. Error bar represents standard deviation (*n* = 3). Differences were detected by Student’s *t*-test (* *p* < 0.05).

After the Calvin cycle, plants synthesize carbohydrates from CO_2_ and water, then store them in their tissues for later use as an energy source or as structural components for internal biochemical reactions. To assess whether increased plant growth and photosynthesis induced by nZVI are associated with alterations in nutrient accumulation in *Arabidopsis*, the selected nutrient contents were measured. Three components of carbohydrates increased significantly in nZVI-exposed plants (Figure 3a): starch content increased by 52%, sucrose increased by 27% and glucose increased by 44%. This increase in the total amount of carbohydrates is plausibly due to increased photosynthesis. Kim et al. observed that the total amounts of soluble sugar and lignin decreased in nZVI-exposed alfalfa roots, but the total amounts in shoots did not differ significantly from the control [40]. The difference from the result observed in *Arabidopsis* may be a consequence of the different phenotypical properties of alfalfa and *Arabidopsis*. Again, plant responses to nZVI with regard to carbohydrate accumulation may vary depending on the plant species.

nZVI did not affect the protein content. Even if the total amount of protein does not differ between groups, the type and function of proteins expressed in each group may be different, so it is necessary to confirm the detailed metabolic process and its mechanism later using omics-based analysis technology.

#### 3.3.2. Mineral Nutrients

Macronutrients such as Mg and P and micronutrients such as Zn and Mn are related to photosynthesis. Therefore, the variations of these four mineral nutrients in *Arabidopsis* were analyzed. nZVI had no obvious influence on Mg uptake, but significantly increased P content and decreased Mn and Zn content in *Arabidopsis* shoots (Figure 3b). It can be assumed that mineral nutrients may also be influenced by nZVI in terms of Fe uptake and the accumulation in *Arabidopsis*. nZVI can thermodynamically reduce Fe solubility by increasing pH by water decomposition [42]. Thus, nZVI can reduce Fe availability in the rhizosphere, thereby stimulating operation of the proton pump in plants. Additionally, nZVI-exposed plants activate PM H^+^-ATPase to extrude protons and acidify their rhizosphere [14]. P is found in soils in both soluble form (H_2_PO_4_^−^ or HPO_4_^2−^) and insoluble form (primary minerals, metal-P complex, and organic P). Plants only take up dissolved P, and since most soil P exists in stable chemical compounds, only a small amount of P is available to plants at any given time [43]. Therefore, the secretion of protons as a result of nZVI-induced H^+^-ATPase activation may increase P availability in soil by acidifying the rhizosphere.

Mn and Zn uptake may also be influenced by Fe uptake. When Fe concentration is increased in the plant, the deposition of Mn and other transition metals can be disrupted [44]. Additionally, Fe and Zn/Mn interact as a consequence of the chemical similarity between their divalent cations and the lack of specificity of the major root iron uptake transporter IRT1 [45]. This phenomenon may control the mutual homeostasis of iron and other mineral nutrients in plants. The presence of nZVI could induce uptake or compete with other nutrient minerals and subsequently result in high or low uptake of a certain mineral nutrient compared to controls. However, these changes did not have a significant effect on plant growth or phenotypes of *Arabidopsis*. Moreover, the uptake of micro- and macronutrients could be affected by a combination of factors, including plant species, soil condition, water deficit and climate [46].

#### 3.3.3. Iron Uptake and Accumulation

**Figure 4:**
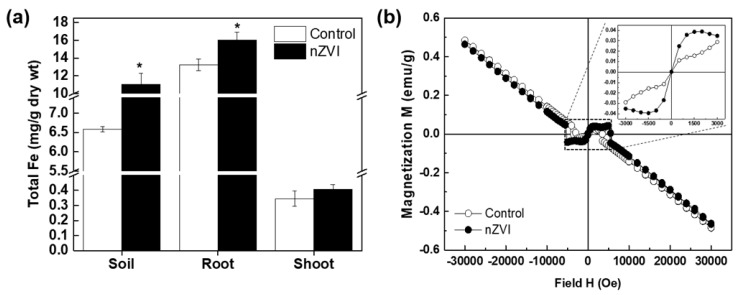
Iron accumulation in soil-grown *Arabidospsis.* (**a**) Fe content in soil, root, and shoots analyzed by inductively coupled plasma optical emission spectrometry (ICP-OES); (**b**) magnetization curves from superconducting quantum interference device (SQUID) analysis of plant roots. Insert of (**b**) shows higher magnification of rectangular regions. Error bar represents standard deviation (*n* = 3). Differences were detected by Student’s *t*-test (* *p* < 0.05).

The Fe content in plant tissues was analyzed to identify nZVI bioavailability. The total Fe concentration was higher in soil and plant tissue (roots and shoots) treated with nZVI than control (Figure 4a). Fe is an essential nutrient for plants, but excessively high Fe accumulation within plant cells can be toxic [47]. Although a high concentration of nZVI (500 mg/kg soil) was used in this experiment, it did not reduce plant growth. A measurable increase was also observed in the Fe concentration of nZVI-treated plant roots compared to the control roots, but the Fe content in nZVI-treated plant shoots was not significantly different from that of the control. Thus, the bioconcentration factor (C_plant_/C_soil_; C = total Fe content) and translocation factor (C_shoot_/C_root_) were lower in nZVI-treated plants than in control plants. In other words, nZVI taken up by roots was poorly translocated to the aerial part of the plant. Our experimental results agree well with the results showing the tendency of Fe accumulation in cucumber cultivated by nZVI-exposed hydroponics [16]. In roots, Fe concentration can be drastically increased by direct contact between the nZVI and fine roots that have a large surface area in the soil [48]. Bioavailable Fe concentration is only slightly higher in nZVI-treated soil than in nontreated soil, whereas the bioavailable Fe concentration of Fe-EDTA–treated soil was more than three times that of nontreated soil [40]. These results indicate that nZVI maintained its particle morphology in the soil and underwent slow oxidation and dissolution. The limited nZVI mobility in soil because of self-aggregation and/or absorption by soil particles and natural organic matter also contributed to the relatively poor Fe translocation and bioaccumulation in shoots [49].

To reconfirm whether nZVI was translocated in the plant, a SQUID analysis was performed to measure the magnetic signals in the plant tissue. Unlike other metal NPs, nZVI has magnetic behavior that can be exploited to study its fate and bioaccumulation in biota [10,25,27,40]. Figure 4b shows the results of the measured magnetization of roots from nZVI-treated *Arabidopsis*. The control sample displayed a diamagnetic property (straight line) that is commonly observed in biological tissues, whereas plant roots treated with nZVI showed weak (super) paramagnetic behavior (S-shaped curve). Ferric citrate, which is a dominant species for chelation and the transportation of Fe in plants, has no magnetization [16]. Therefore, the magnetism found in the roots of nZVI-exposed plants indicates that some magnetic material, not ferric citrate or free ions, accumulated in roots. In contrast, no magnetization signals were detected in shoots (data not shown). Taken together, these results suggest that pristine nZVI was rarely transported from the roots to the aerial part of the plant under these experimental conditions.

**Figure 5:**
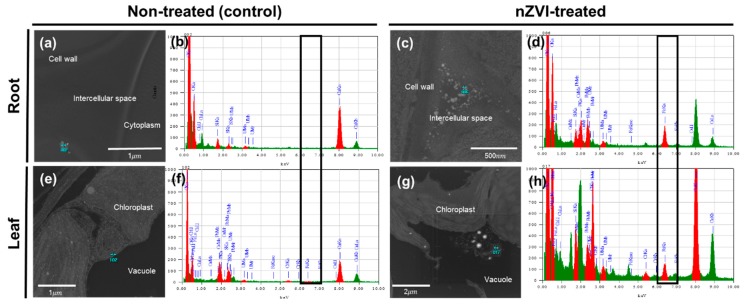
TEM images of (**a**–**d**) root cells and (**e**–**h**) leaf cells of nontreated (control) and nZVI-treated *Arabidopsis.* Blue points in (**a**,**c**,**e**,**h**) were analyzed with EDS, and the spectra are presented in (**b**,**d**,**f**,**h**), respectively. Black boxes in (**b**,**d**,**f**,**h**) show the iron peak in the spectrum.

To investigate whether nZVI could penetrate through the cell walls in the roots and internalize in plant cells, an FE-TEM analysis was performed on root tissue (Figure 5a–d). The sample treated with nZVI showed NPs in the intercellular spaces and surrounding membranes of root cells. The individual NPs were similar in size to pristine nZVI and exhibited aggregation that can be observed in nZVI. TEM coupled with EDS analysis confirmed that these NPs included a certain amount of Fe. These observations suggest that nZVI could penetrate the root cells. nZVI may first contact the root surface and partly enter the pores of the cell wall, then pass through the intercellular space associated with the outer apoplast, without crossing the cell membrane [50,51]. Previous studies have observed NPs or their aggregates in the intercellular space between root cells using TEM analysis, and suggested the existence of an apoplastic pathway for NPs in plant roots (e.g., CuO NPs in rice roots [52], CeO NPs in cucumber roots [53] and ZnO NPs in maize roots [54]). In the upper part, aggregates were deposited in the vacuoles of leaf parenchyma cells. These particles had a different morphology than the NPs that were observed in the roots. An EDS analysis also identified that the aggregate contained Fe that was >100 nm in diameter (Figure 5g). Other circular particles were also found in roots and leaf cells, but they were hundreds of micrometers in size and an EDS analysis determined that their main component was osmium. The most likely explanation is that osmium used for cell staining had condensed within the cell to form a particle structure.

Previous studies found that pristine nZVI was not translocated from the roots to the upper part because of its larger hydrodynamic diameter and this is well matched with our ICP-OES and SQUID analysis results [10,16]. The previous results also suggest that the excess Fe ions precipitate in insoluble forms at the physiological condition in parenchyma cells. Near-edge x-ray absorption fine structure (NEXAFS) and extended x-ray absorption fine structure (EXAFS) measurements identified these precipitated Fe-complexes as iron (oxyhydr)oxides, such as lepidocrocite and maghemite [16]. Another possibility is that Fe could be deposited in the leaf cells as ferritin, which is a ubiquitous protein for Fe storage and sequestration [55]. Since Fe is bound to protein and stored in ionic phase as a soluble, less toxic and bioavailable form, ferritin does not have magnetic behavior, but it can be identified by TEM analysis as particles in leaf cells [56]. In particular, ferritin synthesis is regulated by environmental factors, such as excess Fe or oxidative stress. A consequence of ferritin accumulation in plants is increased H^+^-ATPase activity, which is a key determinant of Fe uptake by dicotyledonous plants such as *Arabidopsis thaliana, Cucumis sativus* and *Solanum lycopersicum* [57]. As a result, the nZVI particles may penetrate the cell walls and enter the plant body. However, the mechanism of uptake and translocation of nZVI into plant tissues is still subject to debate. Only SQUID and TEM-EDS analysis cannot identify the accurate chemical species of particles that were observed in the root tissue. Furthermore, previous nZVI transformation studies were conducted in hydroponics, so the further study of soil-grown plants should be performed using advanced synchrotron radiation-based techniques.

### 3.4. Proposed Mechanism and Implication

**Figure 6:**
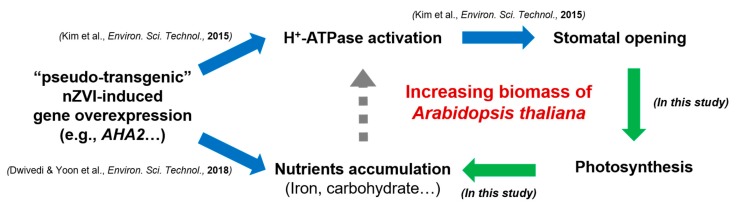
Schematic diagram of proposed plant growth promoting mechanism in this study.

The information from previous studies were combined with the results of this study to propose the following mechanism by which nZVI increases the growth of *Arabidopsis* plants (Figure 6). First, nZVI induced the overexpression of *AHA2* gene, which activates PM H^+^-ATPase in *Arabidopsis thaliana*, thereby promoting stomatal opening [14]. Another study also elucidated the mechanism of nZVI uptake and Fe accumulation in a cucumber plant via the overexpression of *CsHA1* gene, which activates PM H^+^-ATPase in *Cucumis sativus* similar to *AHA2* [16]. In this study, it was found that nZVI-induced stomatal opening increased the photosynthesis of *Arabidopsis*, and the increased photosynthesis led to promoting plant growth, which in turn was associated with the accumulation of nutrients such as soluble sugar, starch and Fe in the plant. There is no direct evidence so far that nutrient accumulation induced by nZVI is associated with H^+^-ATPase activation, but previous studies have shown that enhanced photosynthesis activated by PM H^+^-ATPase causes an accumulation of sugar in plants [58]. Thus, in addition to the existing mechanisms that cause increased plant growth by nZVI by increasing bioavailable Fe, plant hormones and antioxidant enzymes, a new mechanism could be suggested. These results raise the possibility of an ecologically benign alternative approach as a fertilizer or Fe fortifier to increase plant growth. In particular, the use of nano-fertilizer induces the slow release of nutrients from nanoparticles, which increase the uptake efficiency of plants [59]. Additionally, attention should be paid to developing nano-bio technologies for CO_2_ removal from the atmosphere by a pseudo-transgenic plant induced by NPs. For this to be applied, however, further field studies are needed with a wide variety of plant species.

## Figures and Tables

**Table 1:** Characterization of the bed soil used this study.

pH	EC (ds∙m^−1^)	CEC (cmol∙kg^−1^)	TOC (%)	T-N (%)	P (mg∙kg^−1^)	K (mg∙kg^−1^)	Ca (mg∙kg^−1^)	Mg (mg∙kg^−1^)
6.4 ± 0.6	1.99	14.98	1.25	0.120	49 ± 10	53 ± 7	50 ± 8	44 ± 10

EC, electrical conductivity; CEC, cation-exchange capacity; TOC, total organic carbon, T-N: total nitrogen (dry weight basis).

**Table 2:** Gas-exchange parameters of control and nZVI-exposed *Arabidopsis* at 21 days.

Treatment	CO_2_ Assimilation Rate (μmol∙m^−2^∙s^−1^)	Stomatal Conductance (mol∙m^−2^∙s^−1^)	Intracellular CO_2_ Concentration (μL∙L^−1^)	Transpiration Rate (mmol∙m^−2^∙s^−1^)
Control	4.1 ± 0.4 a	0.15 ± 0.02 a	340 ± 4 a	2.1 ± 0.5 a
nZVI	5.2 ± 0.4 b	0.21 ± 0.03 b	348 ± 2 b	3.1 ± 0.3 b

Measurements were conducted at 380 μL∙L^−1^ CO_2_. Results are shown as mean ± standard error (*n* = 6). Letters indicate significant differences between groups (*p* < 0.05 by Student’s *t*-test).
